# 2,4-Dichloro­anilinium 4-chloro­benzene­sulfonate monohydrate

**DOI:** 10.1107/S1600536811012463

**Published:** 2011-04-07

**Authors:** K. Shakuntala, Sabine Foro, B. Thimme Gowda

**Affiliations:** a Department of Chemistry, Mangalore University, Mangalagangotri 574 199, Mangalore, India; b Institute of Materials Science, Darmstadt University of Technology, Petersenstrasse 23, D-64287 Darmstadt, Germany

## Abstract

The asymmetric unit of the title compound, C_6_H_6_Cl_2_N^+^·C_6_H_4_ClO_3_S^−^·H_2_O, contains two 2,4-dichloro­anilinium cations, two 4-chloro­phenyl­sulfonate anions and two water mol­ecules. The three H atoms of the positively charged NH_3_ group have two O atoms of the negatively charged sulfonate anion and one O atom of the water mol­ecule as acceptors. Similarly, the two H atoms of the water mol­ecule have two O atoms of two different negatively charged sulfonate anions as acceptors. Further, one of the O atoms of the sulfonate anion is involved in simultaneous hydrogen bonds with two H atoms, one from the positively charged NH_3_ group and the other from the water mol­ecule. In the crystal, mol­ecules are packed into a layer structure through N—H⋯O(S), N—H⋯O(H_2_O) and N—H⋯O(S)⋯H—O(H_2_O) (three-centre) hydrogen bonding, the chains running along the *c* axis.

## Related literature

For the effect of substituents on the oxidative strengths of *N*-chloro, *N*-aryl­sulfonamides, see: Gowda & Kumar (2003 ▶). For the effect of substituents on the structures of *N*-(ar­yl)-amides, see: Gowda *et al.* (2004 ▶), on *N*-(ar­yl)-methane­sulfonamides, see: Gowda *et al.* (2007 ▶) and on anilinium aryl­sulfonates, see: Shakuntala *et al.* (2011 ▶). For restrained geometry, see: Nardelli (1999 ▶). 
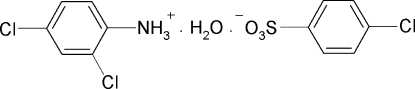


## Experimental

### 

#### Crystal data

C_6_H_6_Cl_2_N^+^·C_6_H_4_ClO_3_S^−^·H_2_O
*M* *_r_* = 372.64
Triclinic, 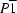

*a* = 7.7589 (8) Å
*b* = 14.143 (2) Å
*c* = 14.358 (2) Å
α = 90.99 (1)°
β = 99.56 (1)°
γ = 90.68 (1)°
*V* = 1553.3 (3) Å^3^
*Z* = 4
Mo *K*α radiation
μ = 0.74 mm^−1^
*T* = 293 K
0.40 × 0.16 × 0.06 mm

#### Data collection

Oxford Diffraction Xcalibur diffractometer with a Sapphire CCD detector
Absorption correction: multi-scan (*CrysAlis RED*; Oxford Diffraction, 2009 ▶) *T*_min_ = 0.757, *T*_max_ = 0.957
11006 measured reflections
6341 independent reflections
2585 reflections with *I* > 2σ(*I*)
*R*_int_ = 0.038

#### Refinement

*R*[*F* ^2^ > 2σ(*F* ^2^)] = 0.051
*wR*(*F* ^2^) = 0.081
*S* = 0.84
6341 reflections
409 parameters
18 restraints
H atoms treated by a mixture of independent and constrained refinement
Δρ_max_ = 0.23 e Å^−3^
Δρ_min_ = −0.25 e Å^−3^

### 

Data collection: *CrysAlis CCD* (Oxford Diffraction, 2009 ▶); cell refinement: *CrysAlis RED* (Oxford Diffraction, 2009 ▶); data reduction: *CrysAlis RED*; program(s) used to solve structure: *SHELXS97* (Sheldrick, 2008 ▶); program(s) used to refine structure: *SHELXL97* (Sheldrick, 2008 ▶); molecular graphics: *PLATON* (Spek, 2009 ▶); software used to prepare material for publication: *SHELXL97*.

## Supplementary Material

Crystal structure: contains datablocks I, global. DOI: 10.1107/S1600536811012463/bq2293sup1.cif

Structure factors: contains datablocks I. DOI: 10.1107/S1600536811012463/bq2293Isup2.hkl

Additional supplementary materials:  crystallographic information; 3D view; checkCIF report

## Figures and Tables

**Table 1:** Hydrogen-bond geometry (Å, °)

*D*—H⋯*A*	*D*—H	H⋯*A*	*D*⋯*A*	*D*—H⋯*A*
N1—H11*N*⋯O1^i^	0.91 (2)	1.89 (2)	2.765 (4)	160 (3)
N1—H12*N*⋯O3^ii^	0.93 (2)	1.83 (2)	2.752 (4)	171 (3)
N1—H13*N*⋯O7	0.90 (2)	1.90 (2)	2.785 (4)	169 (3)
N2—H21*N*⋯O6^iii^	0.91 (2)	1.92 (2)	2.777 (4)	156 (3)
N2—H22*N*⋯O4^iv^	0.93 (2)	1.82 (2)	2.741 (4)	176 (3)
N2—H23*N*⋯O8^iii^	0.91 (2)	1.92 (2)	2.807 (4)	164 (3)
O7—H71*O*⋯O2	0.84 (2)	1.96 (2)	2.778 (4)	163 (4)
O7—H72*O*⋯O6^ii^	0.84 (2)	2.13 (3)	2.879 (4)	148 (5)
O8—H81*O*⋯O1^ii^	0.83 (2)	2.18 (3)	2.900 (4)	146 (4)
O8—H82*O*⋯O5^i^	0.85 (2)	2.03 (2)	2.830 (4)	158 (4)

Symmetry codes: (i) 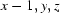; (ii) 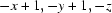; (iii) 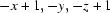; (iv) 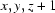.

**Fig. 1.**
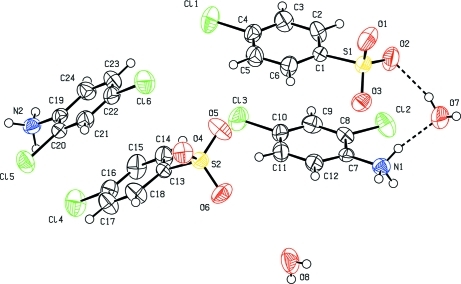
Molecular structure of the title compound, showing the atom-labeling scheme. Displacement ellipsoids are drawn at the 50% probability level.

**Fig. 2.**
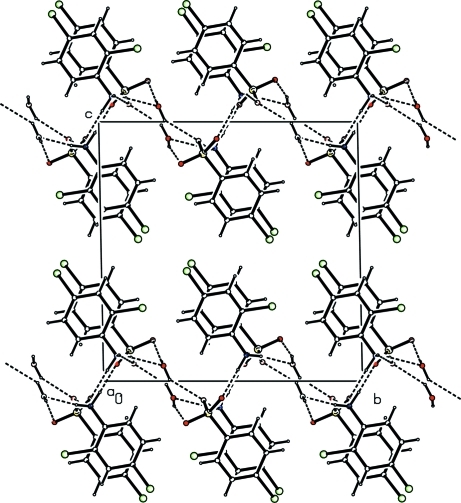
Molecular packing in the title compound. Hydrogen bonds are shown as dashed lines.

## supplementary crystallographic information

### Comment

The amine and sulfonate moieties are important constituents of many important compounds. As a part of studying the substituent effects on the structures of this class of compounds (Gowda & Kumar, 2003; Gowda *et al.*, 2004, 2007; Shakuntala *et al.*, 2011), in the present work, the crystal structure of 2,4-dichloroanilinium, 4-chlorobenzenesulfonate monohydrate (I) has been determined. The compound (I) showed interesting H-bonding in its crystal structure (Fig. 1). It shows 3-centre H-bonding.

The asymmetric unit of (I) contains two 2,4-dichloroanilinium cations, two 4-chlorophenylsulfonate anions and two water molecules. Three H-atoms of the positively charged NH_3_ group have two O atoms of the negatively charged sulfonate anion and one O atom of the water molecule as acceptors. Similarly two H atoms of the water molecule have two O atoms of two different negatively charged sulfonate anions as acceptors. Further, one of the O atoms of the sulfonate anion is involved in simultaneous H-bonding with two H atoms one from the positively charged NH_3_ group of 2,4-dichloroanilinium cation and the other from the water molecule.

The above behavior is in contrast to the N—H···O(S) hydrogen bonding of the three H-atoms of the positively charged NH_3_ group of 2,5-Dichloroanilinium, 4-chlorobenzenesulfonate, having three O atoms of the negatively charged sulfonate anion as acceptors, with each oxygen forming H-bonding with three H-atoms, one each from three positively charged NH_3_ groups (Shakuntala *et al.*, 2011).

The crystal packing of (I) through N1—H11N···O1, N1—H12N···O3, N1—H13N···O7, N2—H21N···O6, N2—H22N···O4 and N2—H23N···O8 hydrogen bonding (Table 1) is shown in Fig. 2.

### Experimental

The solution of chlorobenzene (10 ml) in chloroform (40 ml) was treated dropwise with chlorosulfonic acid (25 ml) at 0° C. After the initial evolution of hydrogen chloride subsided, the reaction mixture was brought to room temperature and poured into crushed ice in a beaker. The chloroform layer was separated, washed with cold water and allowed to evaporate slowly. The residual 4-chlorobenzenesulfonylchloride was treated with 2,4-dichloroaniline in the stoichiometric ratio and boiled for ten minutes. The reaction mixture was then cooled to room temperature and added to ice cold water (100 ml). The resultant title compound (I) was filtered under suction and washed thoroughly with cold water. It was then recrystallized to constant melting point from dilute ethanol.

Rod like colorless single crystals used in X-ray diffraction studies were grown in ethanolic solution by slow evaporation at room temperature.

### Refinement

The H atoms of the NH groups were located in a difference map and later restrained to the distance N—H = 0.89 (2) Å. The H atoms of the water molecules were located in difference map and were refined with restrained geometry (Nardelli, 1999), *viz*. O—H distance was restrained to 0.85 (3) Å and H—H distance was restrained to 1.365 Å, thus leading to the angle of 107 Å. The other H atoms were positioned with idealized geometry using a riding model with C—H = 0.93 Å. All H atoms were refined with isotropic displacement parameters (set to 1.2 times of the *U*_eq_ of the parent atom).

### Crystal data

**Table:**

C_6_H_6_Cl_2_N^+^·C_6_H_4_ClO_3_S^−^·H_2_O	*Z* = 4
*M**_r_* = 372.64	*F*(000) = 760
Triclinic, *P*1	*D*_x_ = 1.593 Mg m^−^^3^
Hall symbol: -P 1	Mo *K*α radiation, λ = 0.71073 Å
*a* = 7.7589 (8) Å	Cell parameters from 2092 reflections
*b* = 14.143 (2) Å	θ = 2.6–27.9°
*c* = 14.358 (2) Å	µ = 0.74 mm^−^^1^
α = 90.99 (1)°	*T* = 293 K
β = 99.56 (1)°	Rod, colorless
γ = 90.68 (1)°	0.40 × 0.16 × 0.06 mm
*V* = 1553.3 (3) Å^3^	

### Data collection

**Table:**

Oxford Diffraction Xcalibur diffractometer with a Sapphire CCD detector	6341 independent reflections
Radiation source: fine-focus sealed tube	2585 reflections with *I* > 2σ(*I*)
graphite	*R*_int_ = 0.038
Rotation method data acquisition using ω and φ scans	θ_max_ = 26.4°, θ_min_ = 2.7°
Absorption correction: multi-scan (*CrysAlis RED*; Oxford Diffraction, 2009)	*h* = −7→9
*T*_min_ = 0.757, *T*_max_ = 0.957	*k* = −17→17
11006 measured reflections	*l* = −16→17

### Refinement

**Table:**

Refinement on *F*^2^	Primary atom site location: structure-invariant direct methods
Least-squares matrix: full	Secondary atom site location: difference Fourier map
*R*[*F*^2^ > 2σ(*F*^2^)] = 0.051	Hydrogen site location: inferred from neighbouring sites
*wR*(*F*^2^) = 0.081	H atoms treated by a mixture of independent and constrained refinement
*S* = 0.84	*w* = 1/[σ^2^(*F*_o_^2^) + (0.0253*P*)^2^] where *P* = (*F*_o_^2^ + 2*F*_c_^2^)/3
6341 reflections	(Δ/σ)_max_ = 0.008
409 parameters	Δρ_max_ = 0.23 e Å^−^^3^
18 restraints	Δρ_min_ = −0.25 e Å^−^^3^

### Special details

**Table:**

Experimental. CrysAlis RED (Oxford Diffraction, 2009) Empirical absorption correction using spherical harmonics, implemented in SCALE3 ABSPACK scaling algorithm.
Geometry. All e.s.d.'s (except the e.s.d. in the dihedral angle between two l.s. planes) are estimated using the full covariance matrix. The cell e.s.d.'s are taken into account individually in the estimation of e.s.d.'s in distances, angles and torsion angles; correlations between e.s.d.'s in cell parameters are only used when they are defined by crystal symmetry. An approximate (isotropic) treatment of cell e.s.d.'s is used for estimating e.s.d.'s involving l.s. planes.
Refinement. Refinement of *F*^2^ against ALL reflections. The weighted *R*-factor *wR* and goodness of fit *S* are based on *F*^2^, conventional *R*-factors *R* are based on *F*, with *F* set to zero for negative *F*^2^. The threshold expression of *F*^2^ > σ(*F*^2^) is used only for calculating *R*-factors(gt) *etc*. and is not relevant to the choice of reflections for refinement. *R*-factors based on *F*^2^ are statistically about twice as large as those based on *F*, and *R*- factors based on ALL data will be even larger.

### Fractional atomic coordinates and isotropic or equivalent isotropic displacement parameters (Å^2^)

**Table:**

	*x*	*y*	*z*	*U*_iso_*/*U*_eq_	
Cl1	1.16798 (13)	0.34833 (8)	0.45361 (8)	0.0716 (4)	
S1	0.83184 (12)	0.59893 (7)	0.12314 (7)	0.0428 (3)	
O1	0.9760 (3)	0.6154 (2)	0.0733 (2)	0.0761 (10)	
O2	0.7727 (3)	0.68469 (18)	0.16210 (19)	0.0660 (8)	
O3	0.6928 (3)	0.54560 (17)	0.06588 (16)	0.0513 (7)	
C1	0.9151 (4)	0.5269 (3)	0.2179 (3)	0.0324 (9)	
C2	0.9687 (4)	0.5667 (3)	0.3058 (3)	0.0473 (11)	
H2	0.9525	0.6308	0.3163	0.057*	
C3	1.0466 (5)	0.5113 (3)	0.3786 (3)	0.0522 (11)	
H3	1.0842	0.5378	0.4384	0.063*	
C4	1.0680 (4)	0.4174 (3)	0.3621 (3)	0.0447 (11)	
C5	1.0119 (5)	0.3772 (3)	0.2755 (3)	0.0539 (12)	
H5	1.0249	0.3127	0.2653	0.065*	
C6	0.9360 (4)	0.4332 (3)	0.2034 (3)	0.0494 (11)	
H6	0.8983	0.4064	0.1438	0.059*	
Cl2	0.38560 (13)	0.65629 (7)	0.28843 (8)	0.0665 (3)	
Cl3	0.65092 (13)	0.32972 (8)	0.42426 (8)	0.0766 (4)	
N1	0.3174 (4)	0.5540 (2)	0.1014 (2)	0.0409 (9)	
H11N	0.207 (3)	0.572 (2)	0.107 (2)	0.049*	
H12N	0.302 (4)	0.520 (2)	0.0444 (17)	0.049*	
H13N	0.370 (4)	0.6097 (16)	0.096 (2)	0.049*	
C7	0.3979 (4)	0.4994 (3)	0.1817 (3)	0.0347 (9)	
C8	0.4360 (4)	0.5398 (3)	0.2709 (3)	0.0386 (10)	
C9	0.5141 (4)	0.4879 (3)	0.3451 (3)	0.0478 (11)	
H9	0.5404	0.5150	0.4053	0.057*	
C10	0.5531 (4)	0.3955 (3)	0.3298 (3)	0.0461 (11)	
C11	0.5190 (4)	0.3543 (3)	0.2417 (3)	0.0494 (11)	
H11	0.5487	0.2918	0.2319	0.059*	
C12	0.4399 (4)	0.4075 (3)	0.1685 (3)	0.0446 (11)	
H12	0.4143	0.3802	0.1083	0.054*	
Cl4	0.59083 (14)	−0.14370 (8)	0.45223 (8)	0.0731 (4)	
S2	0.73669 (12)	0.10495 (7)	0.12039 (7)	0.0406 (3)	
O4	0.8462 (3)	0.05186 (18)	0.06669 (17)	0.0506 (7)	
O5	0.8168 (3)	0.19290 (18)	0.15824 (18)	0.0641 (8)	
O6	0.5644 (3)	0.1171 (2)	0.06778 (19)	0.0699 (9)	
C13	0.7069 (4)	0.0346 (3)	0.2159 (3)	0.0328 (9)	
C14	0.7135 (4)	0.0734 (3)	0.3042 (3)	0.0512 (11)	
H14	0.7418	0.1372	0.3151	0.061*	
C15	0.6781 (5)	0.0181 (3)	0.3773 (3)	0.0569 (12)	
H15	0.6818	0.0445	0.4375	0.068*	
C16	0.6378 (4)	−0.0750 (3)	0.3607 (3)	0.0451 (11)	
C17	0.6327 (5)	−0.1152 (3)	0.2739 (3)	0.0551 (12)	
H17	0.6061	−0.1792	0.2635	0.066*	
C18	0.6676 (5)	−0.0592 (3)	0.2012 (3)	0.0538 (12)	
H18	0.6643	−0.0860	0.1413	0.065*	
Cl5	0.72539 (12)	−0.15530 (7)	0.71771 (7)	0.0605 (3)	
Cl6	0.92906 (13)	0.17163 (8)	0.58487 (8)	0.0729 (4)	
N2	0.7614 (4)	−0.0527 (2)	0.9026 (2)	0.0405 (9)	
H21N	0.644 (2)	−0.062 (2)	0.900 (2)	0.049*	
H22N	0.785 (4)	−0.015 (2)	0.9571 (17)	0.049*	
H23N	0.823 (4)	−0.1072 (16)	0.908 (2)	0.049*	
C19	0.7992 (4)	0.0024 (3)	0.8240 (3)	0.0345 (9)	
C20	0.7885 (4)	−0.0384 (3)	0.7357 (3)	0.0360 (10)	
C21	0.8287 (4)	0.0129 (3)	0.6623 (3)	0.0461 (11)	
H21	0.8226	−0.0147	0.6026	0.055*	
C22	0.8782 (4)	0.1061 (3)	0.6782 (3)	0.0461 (11)	
C23	0.8912 (4)	0.1472 (3)	0.7650 (3)	0.0534 (12)	
H23	0.9274	0.2100	0.7750	0.064*	
C24	0.8500 (4)	0.0946 (3)	0.8383 (3)	0.0472 (11)	
H24	0.8570	0.1224	0.8981	0.057*	
O7	0.4460 (3)	0.7318 (2)	0.0649 (3)	0.0845 (11)	
H71O	0.549 (4)	0.730 (3)	0.095 (2)	0.101*	
H72O	0.450 (5)	0.757 (3)	0.013 (2)	0.101*	
O8	0.0953 (4)	0.2321 (2)	0.0594 (2)	0.0762 (10)	
H81O	0.079 (5)	0.256 (3)	0.007 (2)	0.091*	
H82O	−0.004 (4)	0.225 (3)	0.075 (3)	0.091*	

### Atomic displacement parameters (Å^2^)

**Table:**

	*U*^11^	*U*^22^	*U*^33^	*U*^12^	*U*^13^	*U*^23^
Cl1	0.0821 (8)	0.0756 (9)	0.0568 (8)	0.0228 (6)	0.0064 (6)	0.0255 (7)
S1	0.0377 (6)	0.0442 (7)	0.0456 (7)	0.0034 (5)	0.0033 (5)	0.0096 (5)
O1	0.0391 (15)	0.111 (3)	0.086 (2)	0.0155 (15)	0.0234 (15)	0.063 (2)
O2	0.0791 (19)	0.0450 (16)	0.065 (2)	0.0196 (13)	−0.0149 (15)	−0.0021 (12)
O3	0.0536 (15)	0.0588 (18)	0.0377 (18)	−0.0021 (12)	−0.0035 (11)	0.0008 (12)
C1	0.031 (2)	0.031 (3)	0.034 (3)	0.0002 (18)	0.0031 (17)	0.003 (2)
C2	0.067 (3)	0.032 (3)	0.044 (3)	−0.002 (2)	0.012 (2)	0.003 (2)
C3	0.071 (3)	0.050 (3)	0.034 (3)	−0.003 (2)	0.004 (2)	0.001 (2)
C4	0.043 (2)	0.051 (3)	0.041 (3)	0.004 (2)	0.006 (2)	0.011 (2)
C5	0.067 (3)	0.036 (3)	0.054 (3)	0.006 (2)	0.000 (2)	−0.007 (2)
C6	0.063 (3)	0.038 (3)	0.040 (3)	0.009 (2)	−0.011 (2)	−0.009 (2)
Cl2	0.0937 (8)	0.0391 (7)	0.0702 (9)	−0.0015 (6)	0.0252 (6)	−0.0063 (6)
Cl3	0.0746 (8)	0.0844 (10)	0.0662 (9)	0.0040 (6)	−0.0051 (6)	0.0361 (7)
N1	0.037 (2)	0.045 (3)	0.041 (2)	−0.0020 (17)	0.0088 (19)	0.0030 (19)
C7	0.027 (2)	0.037 (3)	0.039 (3)	−0.0050 (18)	0.0020 (18)	0.009 (2)
C8	0.043 (2)	0.033 (3)	0.041 (3)	−0.0060 (19)	0.012 (2)	−0.003 (2)
C9	0.053 (3)	0.057 (3)	0.033 (3)	−0.011 (2)	0.005 (2)	0.001 (2)
C10	0.041 (2)	0.047 (3)	0.049 (3)	−0.002 (2)	0.002 (2)	0.017 (2)
C11	0.057 (3)	0.038 (3)	0.052 (3)	0.006 (2)	0.003 (2)	0.005 (2)
C12	0.048 (2)	0.038 (3)	0.045 (3)	−0.001 (2)	0.001 (2)	−0.005 (2)
Cl4	0.0945 (8)	0.0798 (9)	0.0496 (8)	0.0056 (7)	0.0221 (6)	0.0264 (7)
S2	0.0369 (6)	0.0439 (7)	0.0414 (7)	−0.0017 (5)	0.0067 (5)	0.0070 (5)
O4	0.0577 (16)	0.0560 (17)	0.0412 (18)	0.0027 (12)	0.0169 (11)	0.0013 (12)
O5	0.0817 (18)	0.0481 (16)	0.068 (2)	−0.0198 (13)	0.0294 (15)	−0.0073 (13)
O6	0.0351 (15)	0.103 (2)	0.072 (2)	0.0003 (14)	0.0030 (14)	0.0520 (19)
C13	0.034 (2)	0.030 (3)	0.034 (3)	−0.0005 (18)	0.0042 (18)	−0.0015 (19)
C14	0.066 (3)	0.043 (3)	0.042 (3)	0.005 (2)	0.003 (2)	−0.003 (2)
C15	0.087 (3)	0.057 (4)	0.026 (3)	0.013 (3)	0.007 (2)	−0.004 (2)
C16	0.051 (2)	0.050 (3)	0.036 (3)	0.007 (2)	0.010 (2)	0.007 (2)
C17	0.078 (3)	0.041 (3)	0.047 (3)	−0.010 (2)	0.014 (2)	−0.001 (2)
C18	0.076 (3)	0.052 (3)	0.035 (3)	−0.015 (2)	0.014 (2)	−0.005 (2)
Cl5	0.0848 (8)	0.0356 (7)	0.0567 (8)	−0.0043 (6)	−0.0006 (6)	−0.0018 (6)
Cl6	0.0835 (8)	0.0727 (9)	0.0622 (8)	−0.0099 (6)	0.0088 (6)	0.0308 (7)
N2	0.0316 (19)	0.044 (3)	0.046 (2)	0.0005 (17)	0.0062 (19)	−0.0010 (19)
C19	0.026 (2)	0.040 (3)	0.038 (3)	0.0024 (18)	0.0059 (18)	0.005 (2)
C20	0.033 (2)	0.032 (3)	0.042 (3)	0.0005 (18)	0.0015 (19)	0.002 (2)
C21	0.053 (3)	0.047 (3)	0.035 (3)	0.002 (2)	−0.001 (2)	0.001 (2)
C22	0.041 (2)	0.049 (3)	0.047 (3)	0.000 (2)	0.004 (2)	0.017 (2)
C23	0.060 (3)	0.036 (3)	0.062 (4)	−0.008 (2)	0.004 (2)	0.005 (3)
C24	0.053 (3)	0.043 (3)	0.044 (3)	−0.006 (2)	0.005 (2)	−0.004 (2)
O7	0.066 (2)	0.069 (2)	0.114 (3)	−0.0011 (18)	−0.002 (2)	0.048 (2)
O8	0.079 (2)	0.062 (2)	0.094 (3)	0.0067 (19)	0.030 (2)	0.0388 (19)

### Geometric parameters (Å, °)

**Table:**

Cl1—C4	1.734 (4)	S2—O5	1.441 (2)
S1—O2	1.439 (3)	S2—O4	1.447 (2)
S1—O3	1.440 (2)	S2—C13	1.752 (4)
S1—O1	1.443 (2)	C13—C18	1.363 (4)
S1—C1	1.754 (3)	C13—C14	1.365 (5)
C1—C6	1.354 (4)	C14—C15	1.382 (5)
C1—C2	1.371 (5)	C14—H14	0.9300
C2—C3	1.380 (5)	C15—C16	1.358 (5)
C2—H2	0.9300	C15—H15	0.9300
C3—C4	1.362 (5)	C16—C17	1.356 (5)
C3—H3	0.9300	C17—C18	1.380 (5)
C4—C5	1.360 (5)	C17—H17	0.9300
C5—C6	1.372 (5)	C18—H18	0.9300
C5—H5	0.9300	Cl5—C20	1.721 (4)
C6—H6	0.9300	Cl6—C22	1.738 (4)
Cl2—C8	1.721 (4)	N2—C19	1.451 (5)
Cl3—C10	1.732 (4)	N2—H21N	0.910 (17)
N1—C7	1.455 (4)	N2—H22N	0.928 (18)
N1—H11N	0.914 (17)	N2—H23N	0.911 (17)
N1—H12N	0.932 (17)	C19—C24	1.360 (4)
N1—H13N	0.896 (18)	C19—C20	1.374 (5)
C7—C12	1.362 (4)	C20—C21	1.366 (5)
C7—C8	1.379 (5)	C21—C22	1.373 (5)
C8—C9	1.366 (5)	C21—H21	0.9300
C9—C10	1.367 (5)	C22—C23	1.354 (5)
C9—H9	0.9300	C23—C24	1.377 (5)
C10—C11	1.367 (5)	C23—H23	0.9300
C11—C12	1.369 (5)	C24—H24	0.9300
C11—H11	0.9300	O7—H71O	0.84 (2)
C12—H12	0.9300	O7—H72O	0.84 (2)
Cl4—C16	1.731 (4)	O8—H81O	0.83 (2)
S2—O6	1.436 (2)	O8—H82O	0.85 (2)
			
O2—S1—O3	112.90 (15)	O6—S2—O4	111.72 (16)
O2—S1—O1	112.42 (19)	O5—S2—O4	112.72 (14)
O3—S1—O1	111.54 (18)	O6—S2—C13	105.10 (15)
O2—S1—C1	107.55 (17)	O5—S2—C13	107.66 (17)
O3—S1—C1	106.54 (16)	O4—S2—C13	106.43 (16)
O1—S1—C1	105.33 (15)	C18—C13—C14	119.3 (4)
C6—C1—C2	119.9 (4)	C18—C13—S2	119.7 (3)
C6—C1—S1	120.3 (3)	C14—C13—S2	120.9 (3)
C2—C1—S1	119.7 (3)	C13—C14—C15	120.1 (4)
C1—C2—C3	119.7 (4)	C13—C14—H14	119.9
C1—C2—H2	120.1	C15—C14—H14	119.9
C3—C2—H2	120.1	C16—C15—C14	119.4 (4)
C4—C3—C2	119.3 (4)	C16—C15—H15	120.3
C4—C3—H3	120.4	C14—C15—H15	120.3
C2—C3—H3	120.4	C17—C16—C15	121.4 (4)
C5—C4—C3	121.2 (4)	C17—C16—Cl4	119.3 (4)
C5—C4—Cl1	119.7 (4)	C15—C16—Cl4	119.3 (3)
C3—C4—Cl1	119.1 (3)	C16—C17—C18	118.7 (4)
C4—C5—C6	118.9 (4)	C16—C17—H17	120.7
C4—C5—H5	120.5	C18—C17—H17	120.7
C6—C5—H5	120.5	C13—C18—C17	121.1 (4)
C1—C6—C5	120.9 (4)	C13—C18—H18	119.5
C1—C6—H6	119.5	C17—C18—H18	119.5
C5—C6—H6	119.5	C19—N2—H21N	111 (2)
C7—N1—H11N	112 (2)	C19—N2—H22N	108 (2)
C7—N1—H12N	113 (2)	H21N—N2—H22N	100 (3)
H11N—N1—H12N	104 (3)	C19—N2—H23N	112 (2)
C7—N1—H13N	114 (2)	H21N—N2—H23N	114 (3)
H11N—N1—H13N	102 (3)	H22N—N2—H23N	111 (3)
H12N—N1—H13N	111 (3)	C24—C19—C20	119.8 (4)
C12—C7—C8	119.3 (4)	C24—C19—N2	119.8 (4)
C12—C7—N1	119.7 (4)	C20—C19—N2	120.5 (4)
C8—C7—N1	121.0 (4)	C21—C20—C19	120.4 (4)
C9—C8—C7	120.2 (4)	C21—C20—Cl5	119.9 (3)
C9—C8—Cl2	120.1 (3)	C19—C20—Cl5	119.7 (3)
C7—C8—Cl2	119.8 (3)	C20—C21—C22	118.9 (4)
C8—C9—C10	119.2 (4)	C20—C21—H21	120.6
C8—C9—H9	120.4	C22—C21—H21	120.6
C10—C9—H9	120.4	C23—C22—C21	121.5 (4)
C11—C10—C9	121.7 (4)	C23—C22—Cl6	119.6 (4)
C11—C10—Cl3	119.3 (4)	C21—C22—Cl6	118.9 (3)
C9—C10—Cl3	119.0 (3)	C22—C23—C24	119.0 (4)
C10—C11—C12	118.2 (4)	C22—C23—H23	120.5
C10—C11—H11	120.9	C24—C23—H23	120.5
C12—C11—H11	120.9	C19—C24—C23	120.5 (4)
C7—C12—C11	121.5 (4)	C19—C24—H24	119.8
C7—C12—H12	119.2	C23—C24—H24	119.8
C11—C12—H12	119.2	H71O—O7—H72O	108 (3)
O6—S2—O5	112.63 (17)	H81O—O8—H82O	107 (3)
			
O2—S1—C1—C6	162.0 (3)	O6—S2—C13—C18	73.4 (3)
O3—S1—C1—C6	40.7 (3)	O5—S2—C13—C18	−166.3 (3)
O1—S1—C1—C6	−77.9 (3)	O4—S2—C13—C18	−45.2 (3)
O2—S1—C1—C2	−21.8 (3)	O6—S2—C13—C14	−103.1 (3)
O3—S1—C1—C2	−143.1 (3)	O5—S2—C13—C14	17.2 (3)
O1—S1—C1—C2	98.3 (3)	O4—S2—C13—C14	138.3 (3)
C6—C1—C2—C3	1.2 (5)	C18—C13—C14—C15	−0.9 (5)
S1—C1—C2—C3	−175.0 (3)	S2—C13—C14—C15	175.6 (3)
C1—C2—C3—C4	−0.5 (5)	C13—C14—C15—C16	0.4 (5)
C2—C3—C4—C5	−0.8 (6)	C14—C15—C16—C17	0.4 (6)
C2—C3—C4—Cl1	179.4 (3)	C14—C15—C16—Cl4	−179.2 (3)
C3—C4—C5—C6	1.4 (6)	C15—C16—C17—C18	−0.7 (6)
Cl1—C4—C5—C6	−178.8 (3)	Cl4—C16—C17—C18	179.0 (3)
C2—C1—C6—C5	−0.7 (6)	C14—C13—C18—C17	0.7 (5)
S1—C1—C6—C5	175.5 (3)	S2—C13—C18—C17	−175.9 (3)
C4—C5—C6—C1	−0.6 (6)	C16—C17—C18—C13	0.1 (6)
C12—C7—C8—C9	−0.4 (5)	C24—C19—C20—C21	0.0 (5)
N1—C7—C8—C9	−178.9 (3)	N2—C19—C20—C21	−178.3 (3)
C12—C7—C8—Cl2	179.3 (3)	C24—C19—C20—Cl5	179.6 (2)
N1—C7—C8—Cl2	0.8 (4)	N2—C19—C20—Cl5	1.3 (4)
C7—C8—C9—C10	−0.3 (5)	C19—C20—C21—C22	−0.6 (5)
Cl2—C8—C9—C10	−180.0 (3)	Cl5—C20—C21—C22	179.8 (2)
C8—C9—C10—C11	1.3 (6)	C20—C21—C22—C23	1.3 (5)
C8—C9—C10—Cl3	−179.6 (2)	C20—C21—C22—Cl6	−179.9 (3)
C9—C10—C11—C12	−1.6 (6)	C21—C22—C23—C24	−1.4 (6)
Cl3—C10—C11—C12	179.3 (3)	Cl6—C22—C23—C24	179.8 (3)
C8—C7—C12—C11	0.0 (5)	C20—C19—C24—C23	−0.2 (5)
N1—C7—C12—C11	178.6 (3)	N2—C19—C24—C23	178.1 (3)
C10—C11—C12—C7	0.9 (5)	C22—C23—C24—C19	0.8 (6)

### Hydrogen-bond geometry (Å, °)

**Table:**

*D*—H···*A*	*D*—H	H···*A*	*D*···*A*	*D*—H···*A*
N1—H11N···O1^i^	0.91 (2)	1.89 (2)	2.765 (4)	160 (3)
N1—H12N···O3^ii^	0.93 (2)	1.83 (2)	2.752 (4)	171 (3)
N1—H13N···O7	0.90 (2)	1.90 (2)	2.785 (4)	169 (3)
N2—H21N···O6^iii^	0.91 (2)	1.92 (2)	2.777 (4)	156 (3)
N2—H22N···O4^iv^	0.93 (2)	1.82 (2)	2.741 (4)	176 (3)
N2—H23N···O8^iii^	0.91 (2)	1.92 (2)	2.807 (4)	164 (3)
O7—H71O···O2	0.84 (2)	1.96 (2)	2.778 (4)	163 (4)
O7—H72O···O6^ii^	0.84 (2)	2.13 (3)	2.879 (4)	148 (5)
O8—H81O···O1^ii^	0.83 (2)	2.18 (3)	2.900 (4)	146 (4)
O8—H82O···O5^i^	0.85 (2)	2.03 (2)	2.830 (4)	158 (4)

Symmetry codes: (i) *x*−1, *y*, *z*; (ii) −*x*+1, −*y*+1, −*z*; (iii) −*x*+1, −*y*, −*z*+1; (iv) *x*, *y*, *z*+1.

## References

[bb1] Gowda, B. T., Foro, S. & Fuess, H. (2007). *Acta Cryst.* E**63**, o2570.

[bb2] Gowda, B. T. & Kumar, B. H. A. (2003). *Oxid. Commun.* **26**, 403–425.

[bb3] Gowda, B. T., Svoboda, I. & Fuess, H. (2004). *Z. Naturforsch. Teil A*, **55**, 845–852.

[bb4] Nardelli, M. (1999). *J. Appl. Cryst.* **32**, 563–571.

[bb5] Oxford Diffraction (2009). *CrysAlis CCD* and *CrysAlis RED* Oxford Diffraction Ltd, Yarnton, England.

[bb6] Shakuntala, K., Foro, S. & Gowda, B. T. (2011). *Acta Cryst.* E**67**, o967. 10.1107/S1600536811010518 PMC3099771 21754230

[bb7] Sheldrick, G. M. (2008). *Acta Cryst.* A**64**, 112–122. 10.1107/S0108767307043930 18156677

[bb8] Spek, A. L. (2009). *Acta Cryst.* D**65**, 148–155. 10.1107/S090744490804362X PMC2631630 19171970

